# Intraoperative radiotherapy as a protocol for the treatment of initial breast cancer

**DOI:** 10.1590/S1679-45082013000400006

**Published:** 2013

**Authors:** Silvio Eduardo Bromberg, Rodrigo de Morais Hanriot, Afonso Celso Pinto Nazário

**Affiliations:** 1 Hospital Israelita Albert Einstein, São Paulo, SP, Brazil; 2 Escola Paulista de Medicina, Universidade Federal de São Paulo, São Paulo, SP, Brazil

**Keywords:** Breast neoplasms/surgery, Breast neoplasms/radiotherapy, Radiotherapy/methods, Antineoplastic protocols

## Abstract

**Objective:**

To report on preliminary outcomes of single-dose intraoperative radiotherapy for early-stage breast cancer based on local recurrence rates and complications.

**Methods:**

Fifty postmenopausal women with ≤2.5cm breast tumors and clinically normal axillary lymph nodes were submitted to quadrantectomy, sentinel lymph node biopsy and intraoperative radiotherapy and studied. Mean follow-up time was 52.1 months.

**Results:**

Mean patient age was 65.5 years; mean tumor diameter was 1.41cm 82% of nodules were hormonal receptor positive and HER-2negative. All patients received a 21 Gy radiation dose for a mean time of 8.97 minutes. Distant metastases were not observed. Local recurrence was documented in three cases, with identical histological diagnosis as the primary tumors. Thirty-five (70%) patients had local fibrosis, with gradual improvement and complete resolution over 18 months. Postoperative infection and seroma formation were not observed.

**Conclusion:**

Partial radiotherapy is a potentially feasible and promising technique. Careful patient selection is recommended before a longer follow-up period has elapsed to confirm intraoperative radiotherapy safety and efficacy.

## RESUMO

**Objetivo:**

Avaliar a experiência inicial de implementação e aplicação de radioterapia única e intraoperatória com feixe de elétrons em pacientes selecionadas com diagnóstico de câncer de mama em estágio inicial. Avaliar também a recorrência local e os eventos adversos (complicações locais).

**Métodos:**

Foram avaliadas 50 pacientes com câncer de mama, pós-menopausadas, com tumores de ≤2,5cm e linfonodos axilares clinicamente não palpáveis, que se submeteram a uma ressecção segmentar e biópsia de linfonodo sentinela e técnica de radioterapia intraoperatória. Essas pacientes foram seguidas por um período médio de 52,1 meses.

**Resultados:**

A idade média dos pacientes foi de 65,5 anos de idade. O diâmetro médio do tumor foi de 1,41cm 82% tinham tumores com receptor hormonal positivo e HER-2 negativo. A dose de radiação empregada foi de 21 Gy em todas as pacientes, com um tempo médio de irradiação intraoperatória de 8,97 minutos. O seguimento médio dessas pacientes foi de 52,1 meses. Foram evidenciados três casos com recorrência local durante esse período, sendo que nenhuma dessas pacientes tinha metástases à distância no momento do diagnóstico da recidiva. O diagnóstico patológico dessas três pacientes foi idêntico ao do tumor primário. Nesta casuística, não se registrou infecção pós-operatória ou formação de seroma. No entanto, em 35 pacientes (70%), foi observada uma fibrose local como sequela de pós-operatório. Esta, quando presente, diminuiu gradualmente e desapareceu completamente em um período médio de 18 meses.

**Conclusão:**

A radioterapia parcial é uma técnica viável e promissora, mas que deve ser indicada em casos selecionados, pelo menos até que tenhamos um maior tempo de seguimento que proporcione maior segurança para indicá-la em nossa rotina, como fazemos com a radioterapia convencional.

## INTRODUCTION

Breast cancer is a global problem accounting for 10.4% of malignant neoplasms in women. Breast cancer is also the fifth leading cause of cancer death^(1)^.

According to data from *Incidência de Câncer no Brasil 2012* (*Instituto Nacional do Câncer - INCA*; HTTP://www1.inca.gov.br/estimativa/2012/), an estimated 50 new breast cancer cases will be diagnosed in 2012. High national breast cancer incidence rates have encouraged the implementation of cancer screening programs for earlier breast cancer diagnosis.

Early-stage breast cancers should be treated with breast-conserving surgery, total or selective (sentinel) axillary lymph node assessment and adjuvant external-beam radiotherapy (EBRT) for improved local-regional control and global survival rates^(2,3)^.

Several independent factors, such as patient age, tumor size, peritumoral vascular invasion, tumor histologic subtype and intracanalicular extension are known to be directly related to cancer recurrence. However, radiotherapy is paramount to decrease local recurrence rates^(3)^. Similar global survival rates have been reported following mastectomy or breast-conserving surgery followed by EBRT^(4-20)^.

The initial hypothesis that wide segmental resection might eliminate the need for EBRT in selected cases was not supported by the outcomes of the B-21trial (National Surgical Adjuvant Breast and Bowel Project - NSABP)^(9)^, which have stressed the relevance of EBRT for decreased recurrence rates. The benefits of adjuvant EBRT to local control and complete breast cancer resolution have been confirmed in randomized studies comparing the outcomes of breast conserving surgery with and without EBRT^(7,10-21)^. According to the National Institutes of Health (NIH, http://www.nih.gov/), breast-conserving surgery followed by EBRT is the current treatment of choice for early-stage breast cancer^(10)^. Sadly, despite widely reported benefits, compliance with adjuvant radiotherapy is not always achieved due to geographical (*e.g.* distance from large cities), socioeconomic and personal constraints (*e.g.* elderly patients requiring support from a caregiver). Also, several countries lack the infrastructure (*e.g.* radiotherapy equipment) required to meet domestic population demands^(11-13)^.

In this scenario, partial breast irradiation seems to be a potentially feasible and practical option. Intraoperative radiotherapy (IORT) with electrons is a partial radiotherapy technique delivering a single radiation dose directly to the tumor bed following quadrantectomy, segmental resection or tumorectomy, with similar biological effectiveness to standard EBRT.

## OBJECTIVE

To report on the preliminary outcomes of breast-conserving surgery followed by IORT in early-stage breast cancer.

## METHODS

This project was approved by the Research Ethics Committee of *Hospital Israelita Albert Einstein* – HIAE (CEP/Einstein number 04/87). Fifty patients were selected out of 104 patients recruited at the HIAE between June 2004 and July 2010. Eligibility criteria were surgical or natural menopause (no menses for more than one year); no previous or concurrent breast cancer treatment; histologic diagnosis of invasive ductal, medullary, colloid (mucinous) or tubular carcinoma without extensive intraductal component and with negative margins upon intraoperative assessment; cancer lesions≤2.5cm in diameter); unilateral breast tumors with no signs of multicentricity or multifocality upon clinical examination, ultrasonography, mammogram and magnetic resonance imaging (MRI) and no previous neoplasm except non-melanoma skin tumors.

Exclusion criteria were as follows: Patients suffering from ductal *in situ,* lobular *in situ,* Paget or inflammatory carcinoma; patients diagnosed with multicentric/multifocal disease based on imaging methods; patients with metastatic tumors at different locations; patients with active collagen disease (particularly systemic lupus erythematosus, scleroderma or dermatomyositis) and comorbidities associated with ≤2 years life expectancy.

Pre-and intraoperative assessment was based on complete clinical history, including family history and tumor detection methods (physical examination, mammogram, ultrasonography and MRI), complete clinical staging (thoracic radiography, abdominal ultrasonography, bone scintigraphy and tumoral markers) and post-operative radiography confirming complete elimination of pre-existing microcalcifications when applicable.

All patients agreed to comply with the treatment plan as discussed with the multidisciplinary team. Informed consent was given in all cases. Patients were operated at the HIAE Radiotherapy Department operating room, next to the room where the linear accelerator employed in this study is located.

Segmental resection was performed under general anesthesia in compliance with surgical oncology guidelines *(e.g. en bloc* tumor resection with broad healthy tissue margins, including deep fascia and skin as necessary). Tumor specimens were submitted to intraoperative histological grading. Unicentricity and surgical margin width were also determined.

Intraoperative histological assessment was performed to confirm the presurgical diagnosis. Surgical margins were marked with china ink to facilitate the determination of margin width. Margins were either curetted for cytology or biopsied (lesions >5mm in size). Biopsy specimens were cut at 4-5mm intervals and evaluated to confirm negative margins (>2mm)^(14,15)^. Additional tissue resection was requested to achieve negative margins as necessary. Lymphadenectomy was performed using the sentinel lymph node method or standard axillary lymph node dissection.

Glandular flaps were mobilized off the subcutaneous tissue and temporarily approximated with sutures to allow IORT to be delivered. The thoracic wall was shielded with a 3mm aluminum-lead disk or similar device to minimize backscattering. Patients were then taken to the adjacent room under general anesthesia and positioned for IORT to achieve alignment between the collimator and the electron beam. Patients were assisted by multidisciplinary team members at all times.

**Figure 1:**
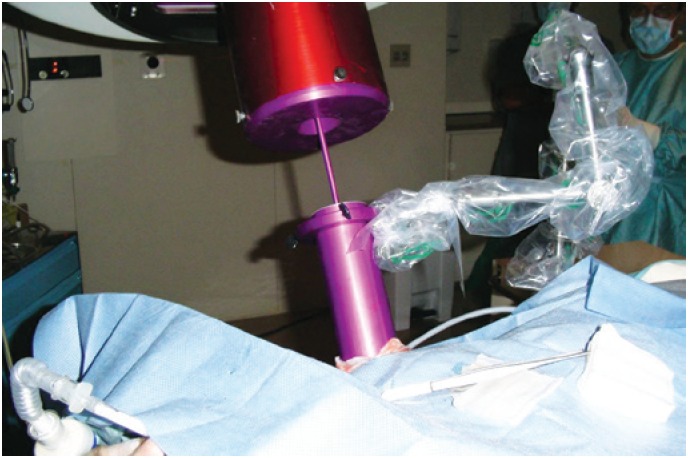
Intraoperative radiotherapy – the collimator (purple) is connected to the linear accelerator and positioned into the mammary gland following elevation of the skin overlying the tumor

**Figure 2:**
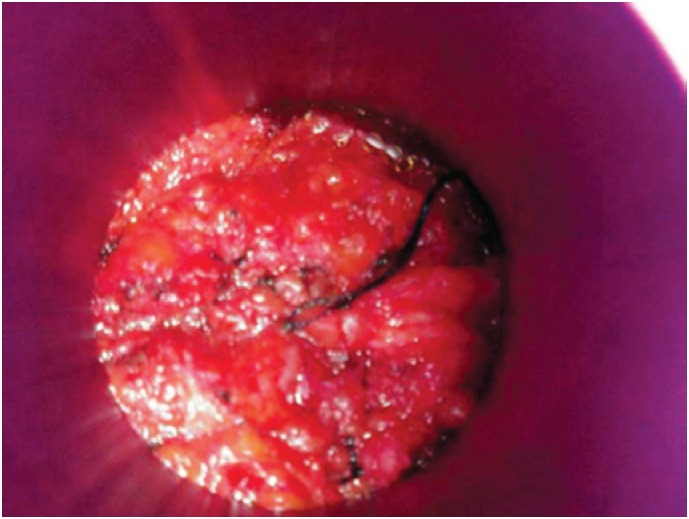
Mammary tissue circumjacent to the tumoral bed following temporary apposition. The target area as defined by the collimator is displayed

Following separation of the skin using a dedicated positioning system, the target area was demarcated using special ink (skin marking pen). A single radiation dose was delivered to the glandular area adjacent to the resected quadrant using a linear particle accelerator (LINAC). Electron energy levels ranged from 6 to 12 Mev according to individual target gland thickness. A full 21Gy dose was delivered using IORT cones and collimators large enough to include wide surgical margins. Energy settings and internal cone diameter and position were determined at the discretion of the medical team (Figures 1 and 2).

IORT was delivered exclusively by radiotherapy team members according to recommendations by Veronesi et al.^(16)^. Previous specific training was provided to the nursery team.

Intra and post-operative histopathological assessment of tumor diameter, tumor histological grade, sentinel and axillary lymph nodes and surgical margins were performed. Tumor histological grade and peritumoral vascular invasion were based on criteria proposed by Elston and Ellis^(17)^ and Rosen et al.^(18)^ respectively. Estrogen (ER) and progesterone (PR) receptor status and Human Epidermal growth factor receptor 2 (HER-2) expression were determined using immunohistochemistry. Fluorescence *in situ* hybridization (FISH)^(19)^ test was employed when necessary.

Hormone therapy with or without combined chemotherapy was recommended based on detailed tumor histology investigation and multidisciplinary case discussion.

Patients with local tumor recurrence were submitted to a second segmental resection or radical mastectomy. Adjuvant therapy was prescribed based on previous treatment outcomes following multidisciplinary case discussion.

Patients were seen by at least one member of the medical team every 3, 4 and 6 months in the first, second and third to fifth follow-up year respectively, and yearly thereafter. Clinical data were thus updated on physicians and radiotherapy records. Mean follow-up time was 52.1 months (median, 54.2 months). All complications were treatment related and consisted of fat necrosis, fibrosis, retraction, deformity or other radiation- dependent events.

## RESULTS

All patients were submitted to adjuvant hormone therapy. Abnormal histopathologic findings (hematoxylin-eosin staining and immunohistochemistry) following singledose IORT were recognized in 7 patients and treated either with chemotherapy or trastuzumab (5 and 2 cases respectively).

All patients studied were submitted to breast-conserving surgery and single-dose IORT. Tumor-free surgical margins were confirmed intraoperatively in all cases. Narrow margins (0.1-0.2mm) were documented in 2 (4%) cases. Affected patients were not reoperated.

Sentinel lymph node investigation was performed in all cases; 2 lymph nodes were removed per surgery on average. Level I and II axillary dissection was performed in 3 patients (6%; 17 lymph nodes removed on average) following recognition of a compromised sentinel lymph node. The sentinel node was confirmed to be the only compromised lymph node in all cases, with compromised areas of less than 2mm.

A single 21 Gy dose of electrons was administered to all patients. Energy levels of 6 MeV and 9 Mev were employed in 27 (54%) and 23 (46%) of patients respectively. Internal cone diameter ranged from 5 to 9cm. Isodose curves (%) were adjusted according to individual gland thickness (Table 1).

**Table 1:** Clinical and radiotherapeuthic data from 50 early-stage breast cancer cases submitted to intraoperative radiotherapy

Variables	Mean	Median	Standard deviation	Minimum	Maximum
Age (years)	65.1	64.0	10.6	45.0	84.0
Tumor diameter (cm)	1.41	1.45	0.54	0.50	2.50
Radiotherapy time (min)	8.97	8.66	1.23	6.88	11.85
Depth (cm)	1.90	1.95	0.46	0.90	3.00
Cone (cm)	4.96	5.10	0.52	4.50	6.40
Isodose curve (%)	97.0	100.0	4.81	85.0	100.0
Follow-up (years)	4.83	4.83	1.60	1.18	7.35

Complications such as post-operative infection, seroma accumulation requiring drainage following suction drain removal, hematomas, wound dehiscence and radiotherapy toxicity were not observed in this study. Local post-operative fibrosis occurred in 35 (70%) patients, with gradual improvement or total resolution over 18 months. Complete fibrosis resolution in less than 12 months was documented in 20 (57.1%) affected patients. Mild skin retraction was observed in 3 (6%) cases. Steatonecrosis without associated clinical signs but evident on palpation and imaging was diagnosed in 5 (10%) patients.

Local recurrence was documented in 3 (6%) patients. Case review revealed tumor-free margins, negative vascular invasion and absence of axillary compromise. Two patients (aged 55 and 61 years respectively) had positive HER-2, ER and PR status and intraparenchymal recurrence at or adjacent to the operated quadrant. The third patient (aged 49 years) had negative HER-2 and positive RE and PR status and recurrence in the surgical scar (non-irradiated area). Histopathologic diagnosis was the same as the primary tumor in all cases.

Local recurrence cases were retreated. Patients with intraparenchymal recurrence were submitted to radical mastectomy and myocutaneous flap reconstruction. The third patient (scar recurrence) was submitted to a second local resection followed by EBRT (50 Gy). All patients progressed well, with no signs of local or distant disease to the time of publication. Distant metastases were not documented and disease-free survival to date was achieved in all cases studied.

## DISCUSSION

Whole-breast EBRT following segmental breast resection is thought to reduce local recurrence rates due to the elimination of local and remote residual neoplasm. This hypothesis is supported by reports of Rosen et al.^(18)^ and Holland et al.^(22)^ on 203 mastectomy specimens: multicentric invasive or *in situ* neoplasm was recognized in 26% specimens with lesions ≤2cm and 36% specimens with lesions measuring between 2.1 and 4cm. However, the biological significance of occult neoplasm and the role of EBRT remain to be determined.

In a meta-analysis (Early Breast Cancer Trialists' Collaborative Group – EBCTCG, 2011) of 17 trials involving 10,081 women with early-stage breast cancer submitted to breast-conserving surgery followed by EBRT, conventional radiotherapy was shown to decrease absolute recurrence rates in patients with negative or positive lymph nodes, and to improve survival rates^(3)^.

The idea of using focal irradiation with partial preservation of the mammary gland was based on local recurrence rate patterns reported in the Milan III trial^(4)^. In that trial, breast tumors measuring less than 2.5cm treated either with quadrantectomy or quadrantectomy and post-surgical EBRT were compared. Reported local recurrence rates (median follow-up period, 119 months) were 21.6% and 5.4% in non-irradiated and irradiated patients respectively (p=0.001).

Locally recurrent lesions following segmental resection and EBRT tend be histologically similar and affect the same site as the primary tumor in 65 to 100% of cases, suggesting the persistence of residual neoplasm in non-irradiated tissues around the operated area^(4,23)^. If local tumor recurrence rates following breast-conserving surgery with negative margins and adjunct EBRT are to be expected in approximately 10% of cases, it could be argued that only 0 to 3.5% of these patients will experience tumor recurrence outside the primary site, a percentage well below the expected incidence of multicentricity reported by Rosen and Holland^(18,24)^.

Several studies investigating the benefits of partial irradiation, respective impacts on breast cancer resolution and applicability in different communities have been published. Different partial radiotherapy techniques are currently being studied with promising, albeit controversial results^(16,25)^.

In some studies, conventional EBRT has been shown to be associated with lower (10%) recurrence rates when compared to partial irradiation (19.5%). However, the opposite outcome was reported in others (*e.g.* non-randomized study by Reitsamer et al.)^(26,27)^.

Intrabeam IORT (a low energy photon beam focused on a specific target area) provides low irradiation energy and is currently being used in the United States, United Kingdom, Germany, Australia and Italy. The first analysis (TARGIT trial)^(25)^ concerning this treatment modality was published in June 2010 and compared the effects of single-dose IORT and EBRT following breast-conserving surgery in early-stage breast cancer. Recurrence and clinically significant toxicity rates of 1% and 3% respectively were reported after a 4-year follow-up^(25)^.

According to the American Society for Therapeutic Radiation Oncology (ASTRO), partial breast radiotherapy is a novel technology for application in selected patients. The superiority of this technique over conventional EBRT in treatment of early-stage breast cancer from a efficacy and safety standpoint remains to be etermined^(28)^.

The IORT modality employed in this study protects the skin from the effects of radiation and enables patients to resume their customary routine activities immediately after recovery from surgery, with less impact on patient personal life. The risk of delayed irradiation due to adjuvant chemotherapy is eliminated and the irradiation of adjacent organs such as the lungs and the contralateral mammary gland is also avoided.

The irradiation method employed in this study was described by Veronesi et al.^(16)^. Ideal radiation dose, applicability, reproducibility and efficacy of the method were investigated in phase I, II and III trials involving 1,822 patients analyzed between 2000 and 2008. Fibrosis without cosmetic disfigurementand liponecrosis were reported in 1.8% and 4.8% of cases, respectively^(16)^. In this study, high local fibrosis rates were documented (70% of patients), with regression over approximately 12 months in 57.1% of cases. Local fibrosis with skin retraction (3 cases; 6%) was the most severe esthetic complication in the cases studied and was graded unimportant by affected patients. Cosmetic repair was therefore not performed.

Contrasting with recurrence rates of 2.3% over 36 months reported by Veronesi et al.^(16)^, a 6% local recurrence rate was documented during the first 42 months following IORT in this study.

Average local recurrence rates of 6.7% have been documented in the first 5 years following breast-conserving surgery and EBRT in patients with negative axillary lymph nodes^(2,3)^. A 15.4% decrease in the absolute local recurrence risk (relative risk, 0.46; 95% confidence interval, 0.41-0.51) has been reported by the ABCTCG in a 10-year follow-up of patients with tumor-free axillary lymph nodes^(3)^.

The high local recurrence rates in this study do not support the efficacy of the treatment modality employed. Despite the low number of patients studied, the importance of strict inclusion criteria and careful patient selection cannot be overemphasized. ASTRO eligibility criteria should be adopted: age ≥60 years, T1 stage, absence of extensive ductal carcinoma *in situ,* negative axillary lymph nodes, ER positive tumors, negative lymphovascular invasion, minimum tumor-free margin width of 2mm and absence of multicentric disease^(29)^.

Despite the above considerations, the fact that all patients studied are alive, without evidences of local or distant disease to the time of publication is encouraging. At this time, IORT should be employed for investigation purposes only. Randomized trials 0413 (Radiation Therapy Oncology Group; RTOG), B-39 (NSABP) and IMPORT LOW (United Kingdom) are expected to clarify IORT indication criteria and efficacy in the near future.

## CONCLUSION

Partial radiotherapy is a feasible and promising technique. However, at this stage strict compliance with ASTRO criteria is recommended and only carefully selected patients should be treated. A longer follow-up period is required before IORT can be considered as safe as conventional radiotherapy.
